# Pharmacotherapy and the risk for community-acquired pneumonia

**DOI:** 10.1186/1471-2318-10-45

**Published:** 2010-07-06

**Authors:** Jen-Tzer Gau, Utkarsh Acharya, Salman Khan, Victor Heh, Lona Mody, Tzu-Cheg Kao

**Affiliations:** 1 Department of Geriatric Medicine/Gerontology, Ohio University College of Osteopathic Medicine (OU-COM), Athens, OH 45701, USA; 2 Department of Internal Medicine, University of South Florida College of Medicine, Tampa, FL 33612, USA; 3 Department of Internal Medicine, Northeastern Ohio University College of Medicine, Akron City Hospital, Akron, OH 44309, USA; 4 Office of Research and Grant, OU-COM, Athens, OH 45701, USA; 5 Geriatrics Research Education and Clinical Center (GRECC), VA Ann Arbor Healthcare System; Division of Geriatrics, University of Michigan, Ann Arbor, MI 48105-2399, USA; 6 Division of Epidemiology and Biostatistics, Department of Preventive Medicine and Biometrics, Uniformed Services University of the Health Sciences, Bethesda, MD 20814, USA

## Abstract

**Background:**

Some forms of pharmacotherapy are shown to increase the risk of community-acquired pneumonia (CAP). The purpose of this study is to investigate whether pharmacotherapy with proton pump inhibitors (PPI), inhaled corticosteroids, and atypical antipsychotics was associated with the increased risk for CAP in hospitalized older adults with the adjustment of known risk factors (such as smoking status and serum albumin levels).

**Methods:**

A retrospective case-control study of adults aged 65 years or older at a rural community hospital during 2004 and 2006 was conducted. Cases (N = 194) were those with radiographic evidence of pneumonia on admission. The controls were patients without the discharge diagnosis of pneumonia or acute exacerbation of chronic obstructive pulmonary disease (COPD) (N = 952). Patients with gastric tube feeding, ventilator support, requiring hemodialysis, metastatic diseases or active lung cancers were excluded.

**Results:**

Multiple logistic regression analysis revealed that the current use of inhaled corticosteroids (adjusted odds ratio [AOR] = 2.89, 95% confidence interval [CI] = 1.56-5.35) and atypical antipsychotics (AOR = 2.26, 95% CI = 1.23-4.15) was an independent risk factor for CAP after adjusting for confounders, including age, serum albumin levels, sex, smoking status, a history of congestive heart failure, coronary artery disease, and COPD, the current use of PPI, β2 agonist and anticholinergic bronchodilators, antibiotic(s), iron supplement, narcotics, and non-steroidal anti-inflammatory drugs. The crude OR and the AOR of PPI use for CAP was 1.41 [95% CI = 1.03 - 1.93] and 1.18 [95% CI = 0.80 - 1.74] after adjusting for the above confounders, respectively. Lower serum albumin levels independently increased the risk of CAP 1.89- fold by decreasing a gram per deciliter (AOR = 2.89, 95% CI = 2.01 - 4.16).

**Conclusion:**

Our study reaffirmed that the use of inhaled corticosteroids and atypical antipsychotics was both associated with an increased risk for CAP in hospitalized older adults of a rural community. No association was found between current PPI use and the risk for CAP in this patient population of our study.

## Background

Community-acquired pneumonia (CAP) is one of the major leading causes of death in older adults [1,2]. In 2006, adults aged 65 years or older accounted for approximately 57% of the total number of pneumonia discharges from hospitals in the US [3].

Identifying risk factors and implementing strategies in reducing the exposure to modifiable conditions may decrease mortality and morbidity. Risk factors that are associated with CAP include age [4,5], chronic obstructive pulmonary disease (COPD) [5], asthma, diabetes mellitus, congestive heart failure [4-6], smoking [4-9], malnutrition [10-13], and aspiration. More studies reveal that some forms of pharmacotherapy may increase the risk of CAP. Current use of atypical antipsychotic drugs is shown to increase the risk of pneumonia in the elderly patients by 2.1 folds in a nested case-control study [14]. The use of proton pump inhibitors (PPI) significantly increases the risk of CAP by 50% to 89% [15-17] and hospital-acquired pneumonia by 30% [18], particularly in those patients with a recent initiation of PPI therapy [15,16,19]. However, a conflicting result was reported with the use of long-term PPI therapy in a study of approximately 880,000 patients by using the UK General Practice Research Database [19]. Subsequently, a meta-analysis study of clinical trials failed to show a definite conclusion [20]. While COPD is increasingly common in older adults, the use of inhaled corticosteroids is shown to significantly increase the risk of pneumonia by 34% as shown by a meta-analysis study (ranges of odds ratio: 0.67-3.16 among the studies cited) [21-25].

Malnutrition contributes to the increasing risks for CAP as demonstrated by several studies [10-13]. Results of previous pharmacotherapy studies that demonstrated the increased risk for CAP were mainly from large data bases, and there was no adjustment for nutritional status among those studies. Because older adults often suffer from malnutrition and therefore, are susceptible to CAP, it is important to include nutritional status in analyzing the association between risk factors and CAP in this age population.

The purpose of this study is to investigate whether the association between pharmacotherapy with PPI, inhaled corticosteroids, and atypical antipsychotics and the increased risk for CAP also holds true among hospitalized older adults of a rural community with the consideration of known risk factors (such as smoking status and serum albumin levels).

## Methods

### Setting

Data were retrieved from 2004 and 2006 medical records of a community hospital by trained research assistants. Medical records from 2005 were not available during the study period (being sent out for microfilm storage). All data were recorded on standardized paper forms and all medical records under study were reviewed and verified by the principal investigator for accuracy. This hospital is the major one in the Athens county community of the State of Ohio that provides 62,000 residents first-line access to an emergency department (ED) and/or hospitalizations. This study was approved by the Ohio University Institutional Review Board.

### Identification and ascertainment of cases and control subjects

Adults aged 65 years or older who were hospitalized during 2004 and 2006 were identified by the assistance of medical record department staff. Patients with the following diagnosis or conditions were excluded from our study: clinically-evident aspiration pneumonia or pneumonitis (N = 47) due to documented dysphagia or with gastro-duodenal tube feeding (N = 7) as patients with aspiration pneumonia differ from CAP in the pathophysiology and risk factors [19]; documented known active lung cancer or metastatic disease (including leukemia; N = 32) as they have underlying conditions that predispose to airway obstruction and pneumonia; being on ventilator therapy (N = 14) as they have a high risk for ventilator-associated and nosocomial pneumonia; death during hospital stay; requiring hemodialysis as those patients were transferred to tertiary care centers; and others (incomplete records or charts not available for review [n = 25]; patients who could not recall medication use [n = 3]).

Cases of CAP were identified initially by the discharge diagnosis and further confirmed by the report of the radiographic findings (a new infiltrate or consolidation) suggestive of pneumonia. Patients with "atypical" clinical presentation (such as mental status changes, increasing lethargy, or accidental falls without the classic symptoms such as productive cough, dyspnea, or fever; N = 17) were included in the final data analysis. Hospital-acquired pneumonia (N = 5) were not included in our study. Patients from nursing homes with pneumonia were included in our data analysis. Only the medical records of the first occurrence of hospitalizations with pneumonia episode during that year were reviewed. Total 194 cases were identified and included in the final data analysis.

The control group was selected as follows: all hospitalized patients aged 65 or older were initially identified by all diagnostic codes (from 0 to 9999). After excluding those with the primary discharge diagnosis of pneumonia, acute and chronic respiratory failure on ventilator therapy, and the conditions previously mentioned in the case group (i.e., death during hospital stay, active lung cancers or metastatic cancers), the first admission medical record was reviewed and data were recorded. Because acute exacerbation of chronic obstructive pulmonary disease (AECOPD) may overlap with pneumonia in clinical presentation, patients with the discharge diagnosis of AECOPD (N = 111) were excluded from both the case and the control groups.

All cases were reviewed by the principal investigator and further efforts were made (based on the documented x-ray findings on admission) to classify each case into an appropriate category (cases vs. controls). The approximate 1:5 ratio of the cases to the controls was not intentional but co-incident. A total of 952 patients were included in the control group.

### Measurement

Age, sex, demographic data, smoking status, past medical history, medication use prior to admission, admission and discharge diagnosis, admission laboratory test results (i.e., white cell count, serum albumin level, and electrolytes) obtained at ED or the first set of blood tests on admission were recorded. Medication use prior to admission was based on the documentation on the medication reconciliation forms or physician's notes (current use vs. non-use). Nursing home residents almost had medication administration records available for review.

PPI is the group of medications that include omeprazole, lansoprazole, pantoprzaole, rabeprazole and esomeprazole. β2 agonist bronchodilator is the group of inhalation medications of short or long-acting effects. Atypical antipsychotic drugs include clozapine, olanzapine, quetiapine, aripiprazole, ziprasidone, and risperidone. Because very few study patients used typical antipsychotic drugs prior to admission, such category of medication was not reported. Systemic corticosteroids were not included in the data analysis as very few patients received such a treatment in our study. Further information on the duration of medication use was not available.

Smoking status was based on the documented ex-smoker vs. current smoker from physicians' notes. Co-morbidity (such as congestive heart failure, coronary artery disease, diabetes, COPD, prior pneumonia, and atrial fibrillation), which is common in the elderly, was the documented previous event or existing condition prior to the study period, and it was recorded as ever vs. never event.

**Missing data **were encountered in the smoking history and laboratory results. Patients without documented data were excluded from the data analysis.

### Data analysis

Mean, standard deviation (SD), and percentage with frequency were used to report continuous and discrete variables. Chi-square test and two-sample t-tests (two-sided) were used to assess if there was a significant association between two groups. Multiple logistic regression with odds ratio (OR) with a 95% confidence interval (CI) was used to measure the association between a risk factor and the occurrence of CAP after adjusting for other variables in the model.

Potential confounders used in the multiple logistic regression model analysis included age, serum albumin levels (gram per deciliter), sex, smoking status (ex- and current smoker), a past medical history of congestive heart failure, coronary artery disease, and COPD, and the use of the following medications: atypical antipsychotic drugs, β2 agonist and anticholinergic bronchodilators, inhaled corticosteroids, and prescribed antibiotic(s) prior to admission, iron supplement, narcotics, and non-steroidal anti-inflammatory drugs (NSAIDs). A history of COPD and medication used for the treatment of COPD were both included in the adjustment in order to analyze whether the treatment is an independent risk factor for CAP in addition to the presence of disease.

Regression diagnostics showed no colinearity among the risk factors and no noticeable outlier or influential observations in the model. Hosmer and Lemeshow goodness-of-fit test was used to assess the fit of the model to data. Statistical significance level was set at a level of .05. Statistical software package, PC SAS version 9.1 (SAS Institute, Inc., Cary, NC) was used to perform the statistical analyses.

## Results

As shown in Table 1, case patients had a significantly different clinical profile compared to the control group. Case patients were more likely to be ex- or current smokers and had a significant medical history of congestive heart failure, coronary artery disease, pneumonia and COPD than the controls. Case patients were more likely to receive atypical antipsychotic drugs, PPI, β2 agonist and anticholinergic bronchodilators, inhaled corticosteroids, prescribed antibiotic(s) prior to admission, narcotics and iron supplements (all p values < 0.05). On the admission blood test results, the case group had a significantly lower mean serum albumin level (2.9 ± 0.5 vs. 3.2 ± 0.5 gm/dL, p < 0.001) and a higher mean white blood cell count (13.6 ± 6.0 vs. 9.3 ± 4.0 K/μL, p < 0.001) than the control group.

**Table 1:** Characteristics of Cases with Community-Acquired Pneumonia and Control Subjects

Variables* N (%)	Cases N = 194	Control N = 952	P value§
Mean age (yr) (SD)	80.3 (8.5)	79.8 (8.1)	.486
Female	119 (61)	641 (67)	.108
White	194 (100)	941 (98.8)	.133
Ex-smoker†	60/191 (31)	151/949 (16)	< .001
Current smoker†	28/191 (15)	73/949 (8)	.002
**Past Medical History**			
Congestive heart failure	63 (32)	201 (21)	< .001
Coronary artery disease	77 (40)	299 (31)	.025
COPD	91 (47)	178 (19)	< .001
Prior pneumonia	38 (20)	43 (5)	< .001
Diabetes mellitus	52 (27)	277 (29)	.520
Atrial fibrillation	35 (18)	136 (14)	.181
Stroke	34 (18)	148 (16)	.492
Cognitive impairment	40 (21)	152 (16)	.114
Depression	58 (30)	243 (26)	.207
Psychiatric illness	7 (4)	24 (3)	.395
GERD	56 (29)	233 (24)	.199
**Medication**			
Atypical antipsychotics	25 (13)	63 (7)	.003
Proton pump inhibitor	86 (44)	343 (36)	.030
H2 receptor antagonist	7 (4)	46 (5)	.460
Antihistamine	29 (15)	129 (14)	.607
β2 agonist bronchodilator	72 (37)	109 (11)	< .001
Inhaled corticosteroid	49 (25)	47 (5)	< .001
Anticholinergic bronchodilator	44 (23)	63 (7)	< .001
Antibiotic use prior to admission	44 (23)	88 (9)	< .001
NSAIDs	27 (14)	115 (12)	.479
Narcotics	60 (31)	224 (24)	.030
Iron supplement	37 (19)	82 (9)	< .001
**Admission laboratory results**			
Serum albumin (gm/dL)	2.9 (0.5) (N = 167)	3.2 (0.5) (N = 807)	< .001
White cell count (K/μL)	13.6 (6.0) (N = 194)	9.3 (4.0) (N = 949)	< .001
Serum potassium (mmol/L)	4.1 (0.6) (N = 194)	4.1 (0.7) (N = 948)	.631
**Discharge diagnoses**			
Congestive heart failure	46 (24)	107 (11)	< .001
Any GI-related illness	18 (9)	269 (28)	< .001
Upper GI illness‡	8 (4)	100 (11)	.006
Lower GI illness¶	10 (5)	140 (15)	< .001
*C. difficile *associated Diarrhea	7 (4)	27 (3)	.564

* Data were presented by number (percent) or mean (SD).

† Smoking status was unknown in 3 patients of cases and in 3 patients of controls.

‡ Including esophageal, stomach, duodenum, and small intestine diagnoses.

¶ Including large colon and rectal diagnoses, but not *C. difficile *associated diarrhea.

§ Obtained using chi-square or two-sample-t test.

Abbreviations: SD = standard deviation; *C. difficile = Clostridium difficile*; COPD = chronic obstructive pulmonary disease; GERD = gastro-esophageal reflux disease; GI = gastro-intestinal; NSAIDs = non-steroidal anti-inflammatory drugs.

Discharge diagnoses among study patients were shown in Additional file 1 and Table 1. There was no difference in the proportions of upper and lower gastro-intestinal (GI) illness, and hepato-biliary-pancreatic diseases between PPI users and non-users (except in the pneumonia diagnosis). While acute congestive heart failure was more often seen in the case group, GI-related diagnoses were more common in the controls (Table 1).

As shown in Table 2, lower serum albumin level, ex- and current smoker, a history of congestive heart failure and COPD, and the use of atypical antipsychotic drugs (adjusted OR = 2.26, 95% CI = 1.23 - 4.15), inhaled corticosteroids (adjusted OR = 2.89, 95% CI = 1.56 - 5.35), and prescribed antibiotic(s) prior to admission (adjusted OR = 1.81, 95% CI = 1.09 - 3.02) was significantly associated with an increased risk for CAP after adjusting for the potential confounders as listed in the table 2. The unadjusted OR of PPI exposure for CAP was 1.41 with 95% CI = 1.03 - 1.93; the association became weaker and not significant after adjusting for all other confounders (adjusted OR = 1.18, 95% CI = 0.80 - 1.74). Our study revealed that lower serum albumin levels independently increased the risk of CAP by 189% by decreasing the concentration of one gram per deciliter (AOR = 2.89, 95% CI = 2.01 - 4.16).

**Table 2:** Odds Ratio for the Risk Factors Associated with Community-Acquired Pneumonia in the Multiple Logistic Regression Model* (N = 969)

**Variables**†	Cases (Yes/No)‡	Control (Yes/No)‡	Crude OR [95% CI]	Adjusted OR [95% CI]
Age (yr)	--	--	1.01 [0.99-1.03]	1.01 [0.99 - 1.04]
Number of serum albumin level below 4 gm/deciliter¶	--	--	3.23 [2.34 - 4.47]	2.89 [2.01 - 4.16]
Sex (female = 1/male = 0)	119/75	641/311	0.77 [0.56 - 1.06]	1.01 [0.66 - 1.53]
Ex-smoker	60/131	151/797	2.42 [1.70 - 3.44]	1.88 [1.15 - 3.06]
Current smoker	28/163	73/876	2.06 [1.29 - 3.29]	2.34 [1.22 - 4.50]
**Past Medical History**				
Congestive heart failure	63/131	201/751	1.80 [1.28 - 2.52]	1.60 [1.04 - 2.46]
Coronary artery disease	77/117	299/653	1.44 [1.05 - 1.98]	1.40 [0.94 - 2.08]
COPD	91/103	178/774	3.84 [2.77 - 5.32]	1.82 [1.17 - 2.82]
**Medication**				
Atypical antipsychotics	25/169	63/889	2.09 [1.28 - 3.41]	2.26 [1.23 - 4.15]
Proton pump Inhibitor	86/108	343/609	1.41 [1.03 - 1.93]	1.18 [0.80 - 1.74]
β2 agonist bronchodilator	72/122	109/843	4.56 [3.21 - 6.50]	1.29 [0.69 - 2.40]
Anticholinergic bronchodilator	44/150	63/889	4.14 [2.71 - 6.31]	1.16 [0.59 - 2.26]
Inhaled corticosteroid	49/145	47/905	6.51 [4.20 - 10.07]	2.89 [1.56 - 5.35]
Antibiotic use prior to admission	44/150	88/864	2.88 [1.93 - 4.30]	1.81 [1.09 - 3.02]
Iron supplement	37/157	82/870	2.50 [1.64 - 3.82]	1.58 [0.94 - 2.67]
Narcotics	60/134	224/728	1.46 [1.04 - 2.04]	1.01 [0.66 - 1.56]
NSAIDs	27/167	115/837	1.18 [0.75 - 1.85]	1.25 [0.70 - 2.23]

* Hosmer and Lemeshow goodness-of-fit test: chi square = 16.43, df = 8, p = .037.

† Variables were dichotomized as 1 = yes, 0 = no, unless stated otherwise.

‡ indicated the number of the presence (yes) and the absence (no) of the medical condition or exposed (yes) vs. unexposed (no) to medication use for the variable of interest.

¶ Which is equal to the number of 4 minus patient's serum level.

Abbreviations: CI = confidence interval; COPD = chronic obstructive pulmonary disease; NSAIDs = non-steroidal anti-inflammatory drugs; OR = odds ratio.

## Discussion

This case-control study reaffirmed that the use of inhaled corticosteroids and atypical antipsychotic drugs was independently associated with an increased risk for CAP among hospitalized older adults of a rural community with the adjustment of nutritional status based on serum albumin levels and other risk factors. However, the association between current PPI exposure and the risk for CAP was not observed in our study. Our study also demonstrated that by decreasing serum albumin level by one gram per deciliter, the risk for CAP can be increased by 1.89- fold in older adults.

COPD has been demonstrated as a major risk factor for CAP. Inhaled corticosteroids, often prescribed for the treatments of COPD and/or asthma, were also identified as an independent risk factor by our study and others [20-24]. Although inhaled corticosteroid therapy may improve the quality of life [26], inappropriate prescriptions of these medications may be highly prevalent among stable COPD patients [27]. Re-evaluating the need for a long-term corticosteroid therapy or using it at a minimally effective dose is warranted.

Our study also demonstrated that atypical antipsychotic drug use independently increased the risk of CAP, which was consistent with the report by Knol et al [14]. Their study included all types of pneumonia in the cases and demonstrated a 2.1-fold increase in the risk for CAP among those taking atypical antipsychotic drugs. With the exclusion of aspiration pneumonia in the case group, our study still revealed a similar result with a modest risk (AOR = 2.26, 95% CI = 1.23-4.15). Effects of atypical antipsychotic drugs on the gastrointestinal system and particularly on the esophageal dysfunction were described [28]. Though it is highly possible that the intrinsically sedative and anticholinergic side effects of this group medication could increase the risk of aspirations by decreasing peristalsis, the exact mechanism of increasing the risk for CAP is not clear.

Our study did not find an association between current PPI therapy and the risk for CAP after the adjustment for confounders. The result was consistent with a recent report of a large nested, case-control study by using the UK General Practice Research Database by Sarkar et al. [19], in which patients with the diagnosis of aspiration pneumonia were excluded in the data analysis as we did in our study. Please note that the study by Sarkar et al. included cancers in the full adjustment when reporting the adjusted OR, while our study excluded active lung cancers and metastatic diseases in the final data analysis. Compared to previous studies that reported the association between PPI use and the risk for CAP (but not hospital-acquired pneumonia) [15-17], the exclusion of aspiration pneumonia was not explicitly mentioned in their studies, which could be one of the possible explanations for the discrepancy of reported findings between ours and other studies.

Our studied patients had a higher prevalence rate of co-morbidities, and a higher percentage of patients received PPI therapy compared to other studies [15-17]. This finding was expected as aging older adults have more co-morbidity and increasingly use pharmacotherapy for chronic illness, especially among hospitalized patients. As the most potent acid-suppression drugs, PPIs have substantially replaced H2 receptor antagonists for the treatment of acid-related gastroesophageal diseases. Evidence suggests that patients with multiple co-morbid conditions are most likely to receive PPI therapy [29], and it is also this group of patients who are most vulnerable to lower respiratory tract infections. Evidence also suggests that hypoalbuminemia and malnutrition are associated with CAP in older adults [10-12], which is also demonstrated by our study. However, PPI users and non-users had no difference in average serum albumin levels among hospitalized older patients as demonstrated in our previous study [30].

Despite PPI therapy drastically reduces gastric acid secretion it does not prevent or inhibit the gastro-esophageal reflux episodes. Clinical observations suggest that reflux of gastric contents into the upper and lower airway may precipitate acute asthma attacks, or acute exacerbation of COPD though there is no strong evidence from clinical studies [31,32]. It is quite possible that PPI therapy could also be prescribed for the early symptoms that are associated with lower respiratory tract infections or gastro-esophageal reflux disease (GERD) (i.e., protopathic bias). This may partially explain why PPI therapy started within the past 7 to 14 days of pneumonia diagnosis was found to be associated with an increased risk for pneumonia in some studies [15,16,19].

Another possible explanation for the different result observed between our and other studies [15,16] was the categorization of exposure history. Other studies [15,16] had data available (based on prescriptions of PPIs) that employed three categories: current, recent, and past use; ours only included current use vs. non-use. Furthermore, our study was hospital-based, case-control. One may suggest that a Berkson's bias, a phenomenon in which the relationship between exposure and disease becomes indistinct in hospital-based studies, may occur in our study. Although we could not exclude the possibility, this current study also demonstrated the association between the use of inhaled corticosteroids and atypical antipsychotic drugs and the increased risk for CAP in our studied patients. Because of the small sample size compared to those population-based studies, the issue of statistical power could be one of the explanations for the lack of the association. However, other investigators [19] of a large study also reported a finding similar to ours in the relationship between overall PPI use and the risk for CAP. Therefore, we do not believe that increasing the sample size will change the conclusions drawn from our study.

Whether age could be an explanation for the discrepancy between our study and the others [15-17] is an interesting question. In the study by Gulmez et al. [16], the association between current use of PPI and CAP became weaker in the age group of older than 60 years (adjusted OR 1.5, 95% CI = 1.3 - 1.7) when compared to the age group of less than 40 years (adjusted OR 2.3, 95% CI = 1.3 - 4.0). We speculate that the association between PPI use and the risk for CAP may become non-significant in the advanced aged group. As demonstrated by Sarkar et al. [19], their paper also reported no association between PPI use and the risk for CAP in persons aged 60 years or older.

As gastric acid production decreases with aging and there is a high prevalence rate of chronic gastritis in people aged older than 70 years, there may be a high prevalence rate of bacterial colonization in the upper gastro-esophageal tract of older adults at baseline even in those older people not receiving PPI therapy. If this is true, use of PPI therapy may not further increase the prevalence rate of bacterial colonization in older adults, by which is the mechanism thought to be responsible for the increased risk for CAP by PPI therapy. There is no information in how PPI therapy affecting the rate of bacterial colonization in older adults compared to young adults in the literature.

One of the strengths in our study was to demonstrate that patients with CAP had a significantly higher mean white blood cell count on admission compared to the control patients (Table 1). Though we could not exclude the possible use of systemic corticosteroid treatment prior to the blood tests obtained in the ED or on admission as the cause of leukocytosis, the exclusion of acute exacerbation of COPD, respiratory failure requiring a ventilator therapy and leukemia cases from our data analysis, as well as very few patients receiving oral systemic steroid therapy, should minimize such a possibility. Our study also demonstrated that lower serum albumin levels independently increased the risk for CAP 1.89-fold in older adults. This is the first of such a report to the best of our knowledge.

The limitations of our study included the lack of diversity as the study subjects were a predominately white population. Therefore, the findings may not apply to other ethnic groups. Furthermore, our data collection resources could not confirm whether patients were compliant with medication use nor the duration as the data collection was based on medical records and physician's notes. Dosage information will provide more insights into the association between a drug therapy and the risk for CAP. Another limitation was unmeasured or residual confounding. Examples are the use of NSAIDs, as readily available over-the-counter, and co-morbidity that may not be completely documented by health-care providers. Certain medical conditions that may be under- assessed and under-documented, such as cognitive impairment or dementia, lack of standardized criteria for making a diagnosis of diseases, as well as the lack of data on cognitive functions prior to and during hospital stay, in which potential limitations are all inherent to a retrospective study design.

## Conclusion

This case-control study of a rural community reaffirmed that the use of inhaled corticosteroids and atypical antipsychotic drugs was an independent risk factor for CAP in hospitalized older adults after adjusting for known risk factors, including serum albumin levels. Current PPI use did not increase the risk for CAP in this hospitalized older population. Our study may imply that other factor(s), such as malnutrition or other pharmacotherapy, may play a greater role than PPI therapy in developing CAP in this population.

## Abbreviations

AECOPD: acute exacerbation of chronic obstructive pulmonary disease; AOR: adjusted odds ratio; CAP: community-acquired pneumonia; CI: confidence interval; COPD: chronic obstructive pulmonary disease; ED: emergency department; GERD: gastro-esophageal reflux disease; NSAIDs: non-steroidal anti-inflammatory drugs; OR: odds ratio; PPI: proton pump inhibitors.

## Competing interests

Drs. Gau, Acharya, Khan, Heh, Mody, and Kao reported no financial conflicts with this research topic and contents.

## Authors' contributions

JTG had full access to all of the data in the study and takes responsibility for the integrity of the data and the accuracy of the data analysis. *Study concept and design*: JTG, TCK. *Acquisition of data*: UA, SK, JTG. *Analysis and interpretation of data*: JTG, VH, UA, SK, LM, TCK. *Preparation of the manuscript*: JTG, UA, SK, VH, LM, TCK. *Statistical analysis*: JTG, VH, TCK. All authors read and approved the final manuscript.

## Authors' Information

JTG and LM are geriatricians. UA and SK are resident physicians in internal medicine. VH is a biostatistician. TCK is a Professor in Epidemiology and Biostatistics.

## Pre-publication history

The pre-publication history for this paper can be accessed here:

http://www.biomedcentral.com/1471-2318/10/45/prepub

## Supplementary Material

Additional file 1 **Appendix Table**. Discharge Diagnoses of Study Patients Based on the Use of Proton-Pump Inhibitors (PPI) Prior to Admission. Click here for file
