# Supplementary material for: Pharmacotherapy and the risk for community-acquired pneumonia
Source: BMC Geriatr. 2010 Jul 6;10:45. doi: 10.1186/1471-2318-10-45 (PMC2909244; doi:10.1186/1471-2318-10-45)
Supplement: Additional file 1 — Appendix Table. Discharge Diagnoses of Study Patients Based on the Use of Proton-Pump Inhibitors (PPI) Prior to Admission. [file 1471-2318-10-45-S1.DOC]

| **Appendix Table: Discharge Diagnoses of Study Patients Based on the Use of Proton-Pump Inhibitors (PPI) Prior to Admission** | | | |
| --- | --- | --- | --- |
| **Discharge diagnosis***  N (%) | **PPI Users** N=429 | **PPI Non-users**  N=717 | **P value§** |
| Community-acquired pneumonia | 86 (20) | 108 (15) | .030 |
| Acute congestive heart failure | 63 (15) | 90 (13) | .304 |
| Any GI-related illness | 112 (26) | 175 (24) | .520 |
| Upper GI illness† | 45 (10) | 63 (9) | .340 |
| Lower GI illness‡ | 60 (14) | 90 (13) | .486 |
| *C*. *difficile* associated  diarrhea | 17 (4) | 17 (2) | .124 |
| Hepato-biliary-pancreatic  illness | 15 (4) | 32 (5) | .425 |
| Any acute fracture | 19 (4) | 38 (5) | .512 |
| Hip fracture | 3 (1) | 11 (2) | .213 |
| Non-hip fracture | 16 (4) | 28 (4) | .881 |

* Indicated number (percent).

† Including esophageal, stomach, duodenum, and small intestine diagnoses.

‡ Including large colon and rectal diagnoses, but not *C*. *difficile* associated diarrhea.

§ Obtained using chi-square.

Abbreviations: *C*. *difficile =* *Clostridium* *difficile*; GI = gastro-intestinal; PPI = proton pump inhibitors.
